# Ugi reaction-derived prolyl peptide catalysts grafted on the renewable polymer polyfurfuryl alcohol for applications in heterogeneous enamine catalysis

**DOI:** 10.3762/bjoc.15.118

**Published:** 2019-06-04

**Authors:** Alexander F de la Torre, Gabriel S Scatena, Oscar Valdés, Daniel G Rivera, Márcio W Paixão

**Affiliations:** 1 Departamento de Química Orgánica, Facultad de Ciencias Químicas, Universidad de Concepción, Edmundo Larenas 234-interior-Casilla 160-C-Concepción, Chile; 2 Márcio W. Paixão, Departamento de Química, Universidade Federal de São Carlos, São Carlos, SP, 97105-900, Brazil; 3 Vicerrectoria de Investigación y Postgrado, Universidad Católica del Maule, Talca 3460000, Chile; 4 Center for Natural Products Research, Faculty of Chemistry, University of Havana, Zapata y G, 10400, La Habana, Cuba

**Keywords:** flow chemistry, heterogeneous catalysis, multicomponent reactions, organocatalysis, polyfurfuryl alcohol

## Abstract

The multicomponent synthesis of prolyl pseudo-peptide catalysts using the Ugi reaction with furfurylamines or isocyanides is described. The incorporation of such a polymerizable furan handle enabled the subsequent polymerization of the peptide catalyst with furfuryl alcohol, thus rendering polyfurfuryl alcohol-supported catalysts for applications in heterogeneous enamine catalysis. The utilization of the polymer-supported catalysts in both batch and continuous-flow organocatalytic procedures proved moderate catalytic efficacy and enantioselectivity, but excellent diastereoselectivity in the asymmetric Michael addition of *n*-butanal to β-nitrostyrene that was used as a model reaction. This work supports the potential of multicomponent reactions towards the assembly of catalysts and their simultaneous functionalization for immobilization.

## Introduction

The immobilization of secondary amine-based catalysts onto organic polymers and silica gel has emerged as an effective strategy that combines the power of heterogeneous and organocatalysis [1–3]. Asymmetric catalysis using polymer-supported chiral organocatalysts usually provides a much greener prospect for the synthesis of enantiomerically enriched building blocks [4–6]. Importantly, immobilized catalysts allow for both recyclability of the catalyst and the implementation of continuous-flow procedures, which usually encompass high reaction yields and reduction of waste – aspects recognized as compatible with the principles of green chemistry [1–5].^.^

However, almost all polymers used in the development of supported organocatalysts are made from non-renewable sources and composed of non-biodegradable materials (e.g., polystyrene) [1–3]. When aiming at implementing large-scale catalytic processes with supported organocatalysts, a relevant “green” premise is the use of renewable and readily available solid supports [1–5]. Accordingly, we envisioned the utilization of the polymer polyfurfuryl alcohol (PFA) – derived from a renewable resource like sugar cane biomass – for the incorporation of chiral pyrrolidine-based motifs capable to catalyze relevant asymmetric reactions. The incorporation of an organocatalyst into a polymer support requires either conjugation to the polymer or functionalization with a polymerizable handle suitable for subsequent copolymerization with a monomeric counterpart. In this regard, multicomponent reactions (MCRs) provide a great opportunity for the simultaneous assembly of the catalyst along with the functionalization polymerizable handle. Orru and co-workers were the first to employ a three-component, diastereoselective variant of the Ugi reaction for the synthesis of a prolyl pseudo*-*peptide catalyst, which proved effective in an organocatalytic conjugate addition reaction [6].

Later, our groups developed an Ugi reaction-based multicomponent approach enabling the structure diversification of prolyl pseudo-peptide catalysts [7], which also proved great efficacy in organocatalytic asymmetric Michael reactions. As extension of this concept to the field of immobilized organocatalysts, we reported the use of the multicomponent approach for the synthesis of silica-grafted peptide catalysts for applications in continuous-flow catalysis [8]. In an endeavor to develop a cheaper and renewable polymer-supported organocatalyst, herein we describe the multicomponent synthesis of furfuryl-containing prolyl pseudo-peptide catalysts and their subsequent utilization in the preparation of PFA-supported catalysts amenable for continuous-flow asymmetric enamine catalysis [9–10].

**Scheme 1:**
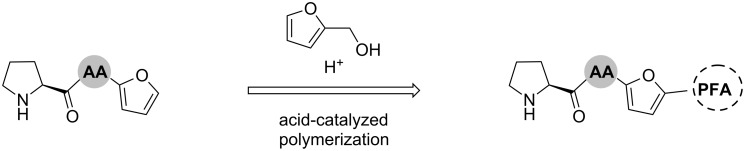
Schematic synthesis of polyfurfulyl alcohol (PFA) incorporating a prolyl peptide catalyst. AA: Amino acid.

The acid-catalyzed polymerization of furfuryl alcohol renders a dark polymer featuring a complex cross-linked polyunsaturated scaffold derived from polycondensation and Diels–Alder reactions [11–12]. Worldwide, there is a well-established industry of furfural production from corncobs and sugarcane pentoses, making polymers derived from this material among the most versatile and promising due to their renewable character and easy exploitation of such biomasses [12–13]. As depicted in Scheme 1, we envisioned the synthesis of prolyl pseudo-peptides having a furan ring handle, which could be subsequently incorporated into PFA during the polymerization process.

## Results and Discussion

**Scheme 2:**
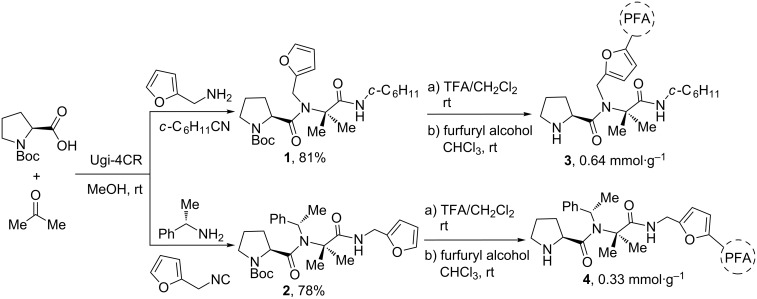
Utilization of the Ugi four-component reaction (Ugi-4CR) for the synthesis of prolyl pseudo-peptide catalysts and their subsequent polymerization with furfuryl alcohol leading to the organocatalytic polymers **3** and **4**. TFA = trifluoroacetic acid; PFA = polyfurfuryl alcohol.

To this end, a solution-phase multicomponent procedure based on the Ugi four-component reaction (Ugi-4CR) [14], was employed to incorporate proline [15] and the furan functionality into pseudo-peptide catalysts. As shown in Scheme 2, Boc-L-proline and acetone were employed as acid and oxo components, respectively, in combination either with furfurylamine and cyclohexyl isocyanide or with (*S*)-α-methylbenzylamine and furfuryl isocyanide. We have previously proven the feasibility of this multicomponent approach for the combinatorial synthesis and rapid screening of pseudo-peptide catalysts [7] and their silica gel-immobilized variants [8]. The choice of using acetone and the *S*-configured α-methylbenzylamine was made in agreement with our previous success with this class of peptide catalyst [7]. In this sense, the corresponding (*R*)-α-methylbenzylamine was not considered because of the good results achieved with the *S*-configured amine, albeit it remains unknown whether there is a match or mismatch between the configuration of the amine and the enantio- and diastereoselectivity of the catalytic process. In this case, furfuryl derivatives – used either as amine or isocyanide component – were ligated [16] to the peptide skeleton aimed at assessing whether the position of the polymerizable handle was important for the organocatalytic performance. Peptides **1** and **2** were subjected to Boc deprotection by treatment with 20% trifluoroacetic acid (TFA) in CHCl_3_ followed by TFA-catalyzed polymerization in the presence of furfuryl alcohol (10 equiv) according to a literature procedure described for PFA [17] (Scheme 2). The polymerization starts as a green solution that eventually turns brown and then black. The polymer suspension was neutralized by washing with a 1 M aqueous solution of NaOH and then precipitated from petroleum ether. The resulting dark solid was ground until the retained material on a 45 µm sieve was less than 10%, thus rendering enough material of PFA-supported prolyl pseudo-peptide catalysts **3** and **4**.

The microanalyses of the polymeric catalysts **3** and **4** show a catalyst loading of 0.64 mmol·g^−1^ and 0.33 mmol·g^−1^, respectively, calculated according to the content of nitrogen by CNHS analysis. The FTIR spectra of polymers **3** and **4** were compared with that of PFA, clearly showing the incorporation of the peptidic moiety into the PFA matrix (see Supporting Information File 1). In detail, the new bands appearing at around 3120 (N–H stretching), 1680 (C=O stretching), 1540 (N–H bending) and 1200 cm^−1^ (CN stretching) are attributed to the typical amide vibrations and are not present in neat PFA. In addition, the bands at around 800, 740 and 600 cm^−1^ confirm the presence of a 2,5-disubstituted furan ring typical of the PFA polymeric matrix [18].

The thermo-oxidative degradation of polymeric catalysts **3** and **4** was also examined using TGA, and compared with that of PFA (see Supporting Information File 1). This analysis showed that both polymers **3** and **4** are quite stable up to 100 °C, but as expected, decompose earlier than neat PFA. Thus, as main difference a significant loss of mass is observed for **3** and **4** at the range of 100–300 °C, whereas neat PFA is still stable at that temperature. This first degradation can be attributed to the decomposition of the peptidic skeleton, while at around 300 °C the decomposition of the polymeric lattice matrix starts, also of PFA. This analysis further demonstrates the incorporation of the peptide moieties into the PFA matrix, while proving the good stability of the PFA-supported catalysts under classic working temperatures (i.e., up to 100 °C).

To assess the catalytic performance of the PFA-supported catalysts, the model system consisting in organocatalytic conjugate addition of *n*-butanal to *trans*-β-nitrostyrene was implemented. During the initial screening, standard reaction conditions comprising the use of 10 mol % of catalyst, toluene as solvent and room temperature were chosen. As shown in Table 1, PFA – used as control – did not afford the Michael product (Table 1, entry 1) due to the lack of the catalytic pyrrolidine moiety. On the other hand, PFA-supported catalysts **3** and **4** gave moderate to good yields depending on the solvent used. In general, polymeric catalyst **3** provided a better yield, enantio- and diastereoselectivity in the Michael adduct than **4** in all tested solvents and conditions.

**Table 1:** Screening of PFA-supported prolyl pseudo-peptide catalysts and the reaction conditions in a batch heterogeneous Michael addition.

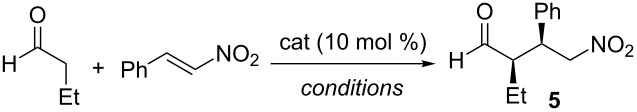					

entry^a^	catalyst	solvent	yield of **5** (%)^b^	dr (*syn*/*anti*)^c^	ee (%)^d^

1	PFA	toluene	–	–	–
2	3	toluene	58	95:5	84
3	4	toluene	52	94:6	29
4	3	THF	83	93:7	77
5	4	THF	62	96:4	53
6	3	acetonitrile	72	94:6	54
7	3	*n*-hexane	54	95:5	56
8^e^	3	*n*-hexane/iPrOH	70	96:4	68
9	3	iPrOH	90	97:3	61
10	3	ethanol	69	96:4	53
11	3	H_2_O	76	96:4	66

^a^All reactions were conducted using 10 mol % of catalyst, 0.25 mmol of β-nitrostyrene and 3 equiv of *n*-butanal in 1 mL of solvent for 24 h. ^b^Yield of isolated pure product. ^c^Determined by ^1^H NMR spectroscopy analysis of the crude product. ^d^Determined by chiral-stationary phase HPLC analysis on the pure product. ^e^9:1 mixture of *n*-hexane*/*isopropanol.

As PFA-supported catalyst **3** proved more effective than **4**, a comprehensive screening of solvents was carried out for the asymmetric Michael addition catalyzed by **3**. This study showed that the yield can be increased up to 90% using isopropanol (Table 1, entry 9), while the diastereoselectivity remains constantly high in all solvents [19]. Unfortunately, the enantioselectivity of the Michael additions remained moderate with both catalysts in all tested solvents and conditions, only rising to 84% ee when using catalyst **3** in toluene. The reason of the better catalytic performance of PFA-supported catalyst **3** compared to **4** may be not only due to higher catalyst loading in the polymer, but also because of the position of the furan ring. During the implementation of the heterogeneous organocatalytic reaction in batch, some polymer features proved limiting the efficiency. For example, the polymer powder showed to be of low density, thus making it difficult to recover the catalysts by decantation. In addition, gravity filtration was employed, however, the powder material mostly remained in the filter paper. To overcome this problem, we turned to implement a continuous-flow organocatalytic system by charging an HPLC column with PFA-supported catalyst **3**. Thus, polymer **3** was packed into a stainless-steel column (Ø = 0.21 cm (diameter), l = 15 cm (length), particle size = 45 µm). The main features of the resulting packed microreactor were determined by pycnometry methodology [20–21], as reported in Table 2.

**Table 2:** Main features of the catalytic microreactor.

loading of **3** (mmol·g^−1^)^a^	amount *w*_tot_ (mg)^b^	*V*_0_ (µL)^c^	*V*_G_ (µL)^d^	*V*_bed_ (µL)^e^	*τ* (min)^f^	ε_tot_^g^

0.639	264	349	519	170	140	0.67

^a^Determined by elemental analysis. ^b^*w*_tot_ = *V*_0_ δ_0_ + *w*_ads_ + *w*_hw_. ^c^*V*_0_ = *w*_1_ − *w*_2_/δ_1_ − δ_2_. ^d^Geometric volume *V*_G_ = π·*h*·*r*^2^·10^3^ (*h* = 15 cm, *r* = 0.105 cm). ^e^*V*_bed_ = *V*_G_ − *V*_0_. ^f^Residence time calculated at flow rate Φ = 2.5 µL·min^−1^, τ = *V*_0_/Φ. ^g^Total porosity ε_tot_ = *V*_0_/*V*_G_.

This method consists in filling the microreactor successively with two distinct solvents (here noted as 1, ethanol and 2, *n*-hexane) and then weighing the filled microreactor accurately. The difference between the masses (*w*) of the filled reactor divided by the differences of solvent densities (δ) permits to calculate the microreactor void volume (*V*_0_, dead volume). This feature is important because it provides an idea of the volume not utilized in the microreactor. The catalyst’s loading was kept as determined by microanalysis, as previously described for polymeric catalyst **3**. The packing amount (*w*_tot_) was also determined by pycnometry. Porosity (ε_tot_) of 0.67 is an optimal value for this material, which is according to the accepted values. One of the most important features of a microreactor for continuous-flow chemistry is the residence time (*τ*) which is known as the time in which a substrate passes through the microreactor without interacting. In some cases, this residence time is measured by the time a dye needs to pass through the reactor. In this work, it was calculated by dividing *V*_0_ by the used flow rate (Φ) of 2.5 µL/min.

**Figure 1:**
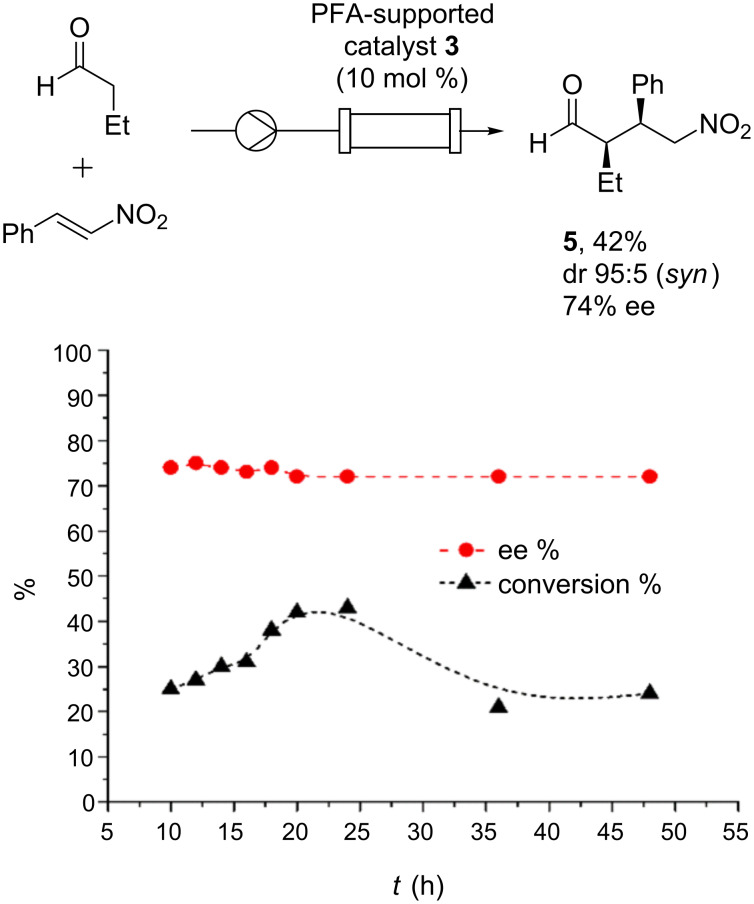
Analysis of the continuous-flow catalytic system producing γ-nitroaldehyde **5** with PFA-supported catalyst **3** packed in a microreactor.

The study of the Michael reaction under continuous-flow conditions started with the optimization of the flow rate. Initially, a solution of β-nitrostyrene (1 equiv, 0.25 M) and *n*-butanal (3 equiv, 0.75 M) in toluene was pumped using a syringe-pump at 2.5 µL·min^−1^ (*τ* = 140 min, Figure 1, top). The concentration was chosen by considering the retention profile of *n*-butanal and β-nitrostyrene in the microreactor. After 22 h, a moderate conversion of the β-nitrostyrene in toluene was observed, proving a poor efficiency of the process and a low value of productivity. This clearly showed that the conversion of the starting material is increasing until 24 h, and after that, the reactor productivity decreases considerably (Figure 1, bottom). We hypothesized that the higher residence time of β-nitrostyrene in the reactor may lead to a lower yield or the catalyst may acquire inactivation. Accordingly, all the substrates were injected into the reactor coupled to a HPLC system and the retention times of each substrate within the reactor were measured by UV detection at a wavelength of 210 nm. A retention time of 70 min for β-nitrostyrene at a flow rate of 0.1 mL/min into the reactor was observed. Then, this preferential occupancy of the packing material by β-nitrostyrene (50 times as residence time calculated) limits the formation of the Michael product and, consequently, lowers the chemical efficiency. Despite the good level of diastereocontrol in Michael addition (dr 95:5 *syn*/*anti*), a little drop in the enantioselectivity was observed (i.e., 74% ee) compared to the batch process with the same catalyst. Nonetheless, the enantioselectivity remains constant during the whole time of experiment, as shown in Figure 1. Finally, the overall yield of isolated Michael adduct **5** was 42% after column chromatography, which is in agreement with the conversion determined during the continuous-flow study.

## Conclusion

In conclusion, we have implemented a multicomponent approach for the one-pot assembly of furfuryl-containing organocatalysts suitable for the incorporation into a polyfurfuryl polymer. Two polymer-supported prolyl peptide catalysts were produced by means of an initial Ugi reaction followed by an acid-catalyzed polymerization. They catalytic polymers were screened in the heterogeneous catalytic Michael addition in batch, proving that catalyst **3** is more effective and provides better stereoselectivity. A continuous-flow organocatalytic system was also implemented using catalyst **3**, enabling the continuous production of a γ-nitroaldehyde in moderate yield and enantioselectivity, but with excellent diastereoselectivity.

## Experimental

^1^H NMR and ^13^C NMR spectra were recorded at 400 MHz for ^1^H and 100 MHz for ^13^C, respectively. Chemical shifts (δ) are reported in parts per million relative to the residual solvent signals, and coupling constants (*J*) are reported in hertz. Flash column chromatography was carried out using silica gel 60 (230–400 mesh) and analytical thin-layer chromatography (TLC) was performed using silica gel aluminum sheets. HPLC chromatograms were obtained on a Shimadzu apparatus, LC-10AT Pump, SPD-10A UV–vis detector, SCL-10A system controller, using a Chiralpak AD-H (4.6 mm Ø × 250 mm length, particle size 5 μm). Optical rotations were measured at the indicated temperature using a Perkin-Elmer Polarimeter, Mod. 241, (wavelength: 589 nm). Melting points were obtained in a MQAPF-301 apparatus.

**General procedure A.** A suspension of the amine (1.0 mmol) and acetone (1.0 mmol) in MeOH (5 mL) was stirred for 1 h at room temperature. The carboxylic acid (1.0 mmol) and the isocyanide (1.0 mmol) were then added and the reaction mixture was stirred at room temperature for 24 h. The volatiles were removed under reduced pressure and the resulting crude product was dissolved in 100 mL of CH_2_Cl_2_. The organic phase was washed sequentially with an aqueous saturated solution of citric acid (50 mL), aqueous 10% NaHCO_3_ (50 mL), and brine (50 mL), and then dried over anhydrous Na_2_SO_4_ and concentrated under reduced pressure.

**General procedure B.** The prolyl pseudo*-*peptides catalyst was dissolved in 3 mL of CH_2_Cl_2_ and treated with 1 mL of trifluoroacetic acid at 0 °C. The reaction mixture was allowed to reach room temperature, stirred for 4 h and then concentrated to dryness (the excess of TFA was removed by repetitive addition and evaporation of further CH_2_Cl_2_). The crude product was re-dissolved in 10 mL of CHCl_3_ for the polymerization step. To a suspension of the salt pseudo-peptide catalysts (1.0 mmol, 1 equiv) and furfuryl alcohol (10 mmol, 10 equiv) in CHCl_3_ (5 mL) was added TFA (0.5 mmol, 5 mol %) dropwise over 10 min, and stirred for 24 h at room temperature. The color of the solution changed during the reaction from yellow-green to brown then black. The neutralization of the polymerization solution was carried out with a concentrated basic solution. The use of a 1 M NaOH (5 mL) solution requires two washes of 10 min each but at the end of the reaction an emulsion may appear. In order to avoid this problem an excess of 0.1 M NaOH solution was used. Polymers were isolated by precipitation in petroleum ether and dried in high vacuum. The resulting dark solid was ground until the retained material on a 45 µm sieve was lower than 10%.

**PFA:** For comparison, PFA was prepared in a conventional way according to a reported procedure [17].

**General procedure C.** The nitroolefin (0.25 mmol, 1.0 equiv) and the aldehyde (0.75 mmol, 3.0 equiv) were added to a solution of the prolyl pseudo-peptide catalyst (0.025 mmol, 0.01 equiv) in the solvent of choice (1 mL). The reaction mixture was stirred for 24 h and then concentrated under reduced pressure. The resulting crude product was purified by flash column chromatography on silica gel using *n*-hexane/EtOAc as eluent. Enantiomeric excess (ee) was determined by chiral HPLC analysis through comparison with the authentic racemic material. Assignment of the stereoisomers was performed by comparison with literature data.

### Synthesis and characterization

**Prolyl pseudo*-*peptide 1.** Furfurylamine (177 µL, 2 mmol), acetone (116 mg, 2 mmol), Boc-L-Pro-OH (431 mg, 2 mmol) and cyclohexyl isocyanide (249 µL, 2 mmol) were reacted in MeOH (5 mL) according to the general procedure A. Flash column chromatography purification (EtOAc/hexane 1:1, v/v) afforded the Boc-proline-based peptide **1** as colorless oil. A mixture of conformers was observed by NMR (ratio 3:1). Assigned signals belong to the mixture of conformers. Yield: 81%; *R*_f_ 0.34 (EtOAc/hexane 1:1, v/v); [α]_D_^20^ − 19.9 (*c* 0.0085 g·cm^−3^, MeOH); ^1^H NMR (400 MHz, CDCl_3_) δ 0.99–1.19 (m, 3H), 1.29–1.39 (m, 2H), 1.43 (s, 3H), 1.45 (s, 9H), 1.48 (s, 3H), 1.58–2.01 (m, 9H), 2.10 (m, 1H), 3.39 (m, 1H), 3.53 (m, 1H), 3.65 (m, 1H); 4.50, 4.52 (2×d, *J* = 16.0 Hz, 1H), 4.60 (m, 1H), 4.77, 5.09 (2×d, *J* = 18.2 Hz, 1H), 5.70, 5.94 (2×d, *J* = 7.2 Hz, NH, 1H), 6.39 (m, 1H), 7.40 (d, *J* = 7.8 Hz, 1H); ^13^C NMR (100 MHz, CDCl_3_) δ 23.1, 24.2, 24.4, 24.9, 25.1, 25.5, 28.6, 30.2, 32.7, 32.8, 41.5, 47.2, 48.4, 56.9, 63.3, 79.5, 107.3, 110.8, 141.9, 152.2, 154.7, 173.7, 174.1.

**Prolyl pseudo-peptide 2.** (*S*)-(−)-α-Methylbenzylamine (257 µL, 2 mmol), acetone (147 µL, 2 mmol), Boc-L-Pro-OH (431 mg, 2 mmol) and furfuryl isocyanide (216 µL, 2 mmol) were reacted in MeOH (5 mL) according to the general procedure A. Flash column chromatography purification (EtOAc/hexane 1:1, v/v) afforded the proline-based peptide **2** as colorless oil. Yield: 78%; *R*_f_ 0.30 (EtOAc/hexane 1:1, v/v); [α]_D_^23^ −6.26 (*c* 0.0047 g·cm^−3^, MeOH); ^1^H NMR (400 MHz, CDCl_3_) δ 1.40 (s, 9H), 1.41–1.75 (m, 9H), 1.94 (m, 3H), 3.26–3.37 (m, 2H), 4.08–4.11 (m, 2H), 4.59–4.65 (m, 1H), 6.23–6.29 (m, 2H), 7.26–7.40 (m, 4H), 7.53 (m, 2H); ^13^C NMR (100 MHz, CDCl_3_) δ 19.2, 24.2, 24.4, 26.7, 28.9, 37.2, 47.7, 51.9, 59.3, 64.8, 79.5, 106.4, 110.4, 127.4, 128.9, 141.3, 142.8, 152.9, 154.8, 175.4, 175.5.

**PFA-supported catalyst 3**. Compound **1** (476 mg, 1 mmol, 1.0 equiv), furfuryl alcohol (860 µL, 10 mmol, 10 equiv) and TFA (38 µL, 0.5 mmol) were reacted in CHCl_3_ (5 mL) according to the general procedure B. After precipitation in petroleum ether, polymer **3** was obtained as a black amorphous solid. IR (KBr, cm^−1^): 3500, 3120, 2930, 2860, 1720, 1680, 1540, 1420, 1320, 1180, 1080, 790. 740, 600; microanalysis: N (2.68%), C (58.29%), H (5.12%), S (0%); loading = 0.64 mmol·g^−1^.

**PFA-supported catalyst 4.** Compound **2** (545 mg, 1 mmol, 1.0 equiv), furfuryl alcohol (860 µL, 10 mmol, 10 equiv) and TFA (38 µL, 0.5 mmol) were reacted in CHCl_3_ (5 mL) according to the general procedure B. After precipitation in petroleum ether, polymer **4** was obtained as a black amorphous solid. IR (KBr, cm^−1^): 3500, 3120, 2930, 1720, 1680, 1610, 1550, 1420, 1350, 1200, 1160, 1110, 1038, 780, 740, 600; microanalysis: N (1.36%), C (50.52%), H (3.77%), S (0%); loading = 0.33 mmol·g^−1^.

**PFA.** Furfuryl alcohol (860 µL, 10 mmol) and TFA (38 µL, 0.5 mmol) were reacted in CHCl_3_ (5 mL) according to the general procedure B. After precipitation in petroleum ether, PFA was afforded as a black amorphous solid. IR (KBr, cm^−1^): 3480, 2930, 1718, 1420, 1150, 1100, 800, 690, 600; microanalysis: N (0 %), C (56.14%), H (4.10%), S (0%); loading of catalyst = 0 mmol·g^−1^

(2*R*,3*S*)-2-Ethyl-4-nitro-3-phenylbutanal (**5**). Prepared by reaction of *n*-butanal with *trans*-β-nitrostyrene according to the general procedure C. The compound was purified by flash column chromatography (EtOAc/hexane 1:9, v/v). The spectroscopic data are in agreement with the published data [15]. The enantiomeric excess was determined by chiral-stationary phase HPLC (Chiralpak OD-H, hexane/iPrOH 99:1, v/v, 25 °C) at 1.00 mL/min, UV detection at 210 nm: *t*_R_: (*syn*, major) = 28.4 min, (*anti*, minor) = 20.9 min; *R*_f_ 0.26 (EtOAc/hexane 2:8, v/v); [α]_D_^23^ +25.21 (*c* 0.0046 g·cm^−3^, MeOH); ^1^H NMR (400 MHz, CDCl_3_) δ 9.72, 9.49 (2×d, *J* = 2.6 Hz, 1H, CHO), 7.36–7.29 (m, 3H, Ph), 7.19–7.17 (m, 2H, Ph), 4.72 (dd, *J* = 5.0 Hz, 12.7 Hz, 1H, CH_2_NO_2_), 4.63 (dd, *J* = 9.6 Hz, 12.7 Hz, 1H, CH_2_NO_2_), 3.79 (td, *J* = 5.0 Hz, 9.8 Hz, 1H, CHPh), 2.71–2.65 (m, 1H, CHCHO), 1.54–1.47 (m, 2H, CH_2_CH_3_), 0.83 (t, *J* = 0.83 Hz, 3H, CH_3_); ^13^C NMR (100 MHz, CDCl_3_) δ 203.2, 136.8, 129.1, 128.1, 128.0, 78.5, 55.0, 42.7, 20.4, 10.7.

**Preparation of microreactor column.** PFA-supported catalyst **3** (500 mg, excess, suspended in 25 mL of ethanol) was packed into a stainless-steel HPLC column (Ø = 2.1 mm, l = 150 mm, particle size ≤ 45 µm). The packing was performed under constant pressure (2500 psi) using ethanol (250 mL) as the solvent by using an air-driven liquid pump.

## Supporting Information

File 1 ^1^H and ^13^C NMR spectra of prolyl pseudo-peptide catalysts and chiral-phase HPLC analysis of Michael adducts.
