# Supplementary material for: Ugi reaction-derived prolyl peptide catalysts grafted on the renewable polymer polyfurfuryl alcohol for applications in heterogeneous enamine catalysis
Source: Beilstein J Org Chem. 2019 Jun 4;15:1210–6. doi: 10.3762/bjoc.15.118 (PMC6604708; doi:10.3762/bjoc.15.118)
Supplement: File 1 — 1H and 13C NMR spectra of prolyl pseudo-peptide catalysts and chiral-phase HPLC analysis of Michael adducts. [file Beilstein_J_Org_Chem-15-1210-s001.pdf]

BEILSTEIN JOURNAL OF ORGANIC CHEMISTRY

Supporting Information

for

Ugi reaction-derived prolyl peptide catalysts grafted on the renewable polymer polyfurfuryl alcohol for applications in heterogeneous enamine catalysis

Alexander F. de la Torre, Gabriel S. Scatena, Oscar Valdés, Daniel G. Rivera and Márcio W. Paixão

Beilstein J. Org. Chem. **2019,** *15,* 1210–1216. doi:10.3762/bjoc.15.118

^{1}H and ^{13}C NMR spectra of prolyl pseudo-peptide catalysts and chiral-phase HPLC analysis of Michael adducts

License and Terms: This is a supporting information file under the terms of the Creative Commons Attribution License (http://creativecommons.org/licenses/by/4.0). Please note that the reuse, redistribution and reproduction in particular requires that the authors and source are credited.
The license is subject to the *Beilstein Journal of Organic Chemistry* terms and conditions: (https://www.beilstein-journals.org/bjoc)

Spectra of compounds

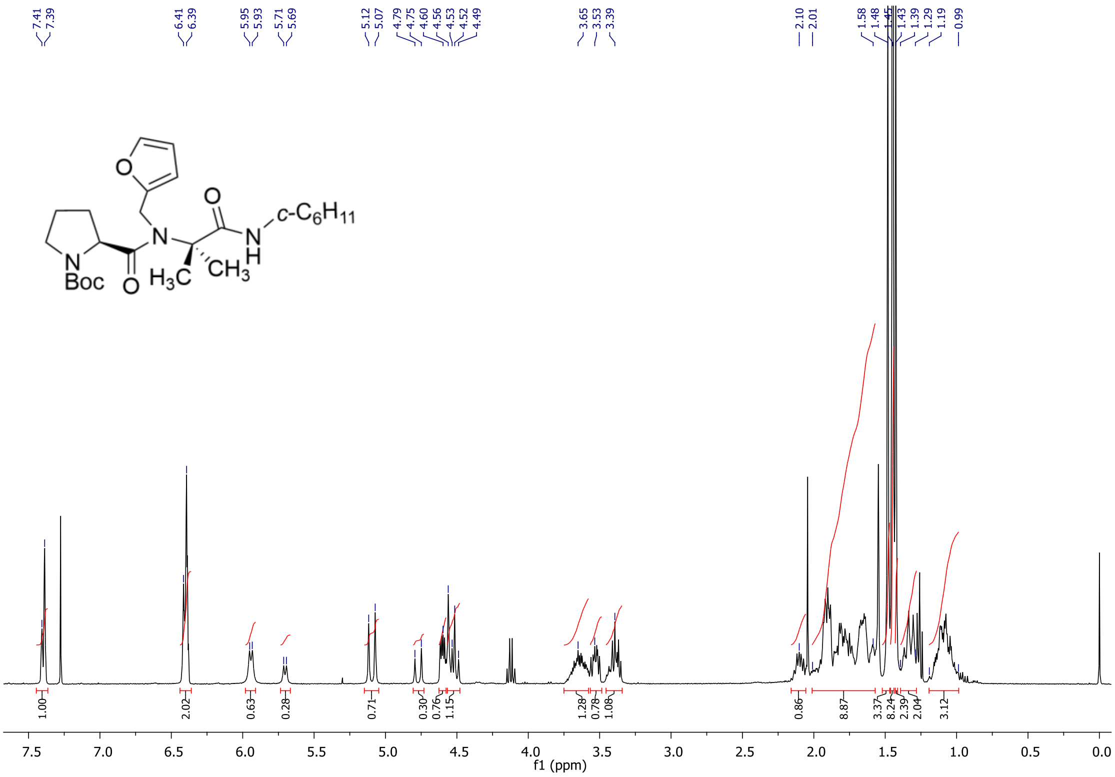

Figure S1: 400 MHz ^{1}H NMR spectrum in $CDCl_3$ of **1**.

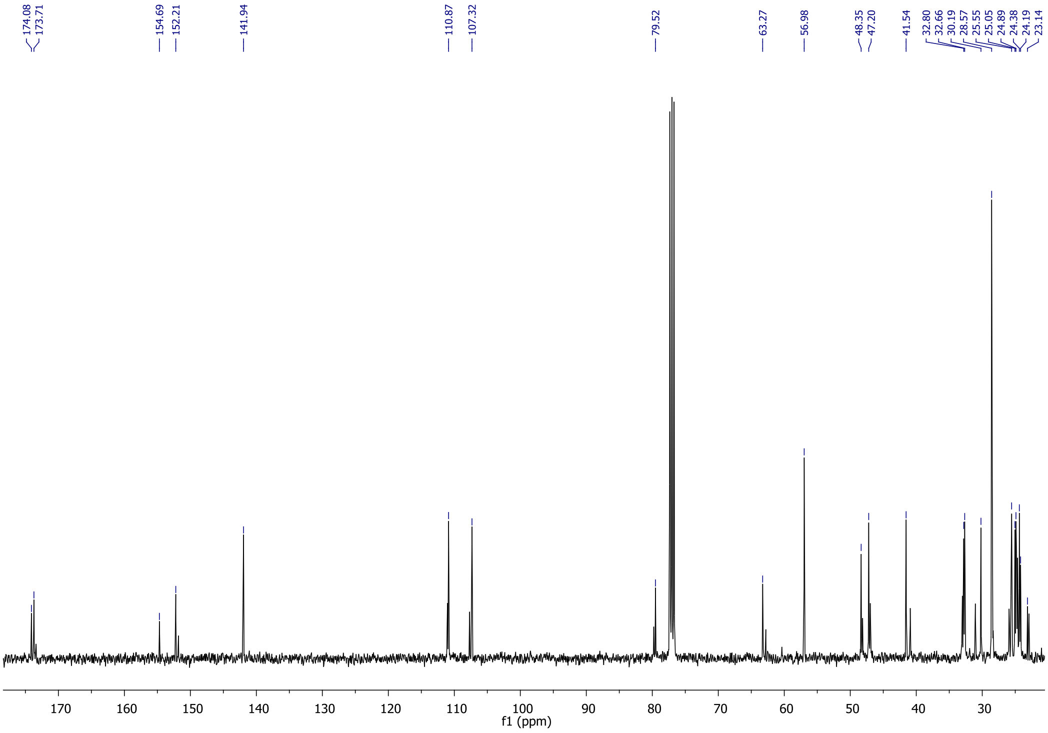

Figure S2: 100 MHz ^{13}C NMR spectrum in $CDCl_3$ of **1**.

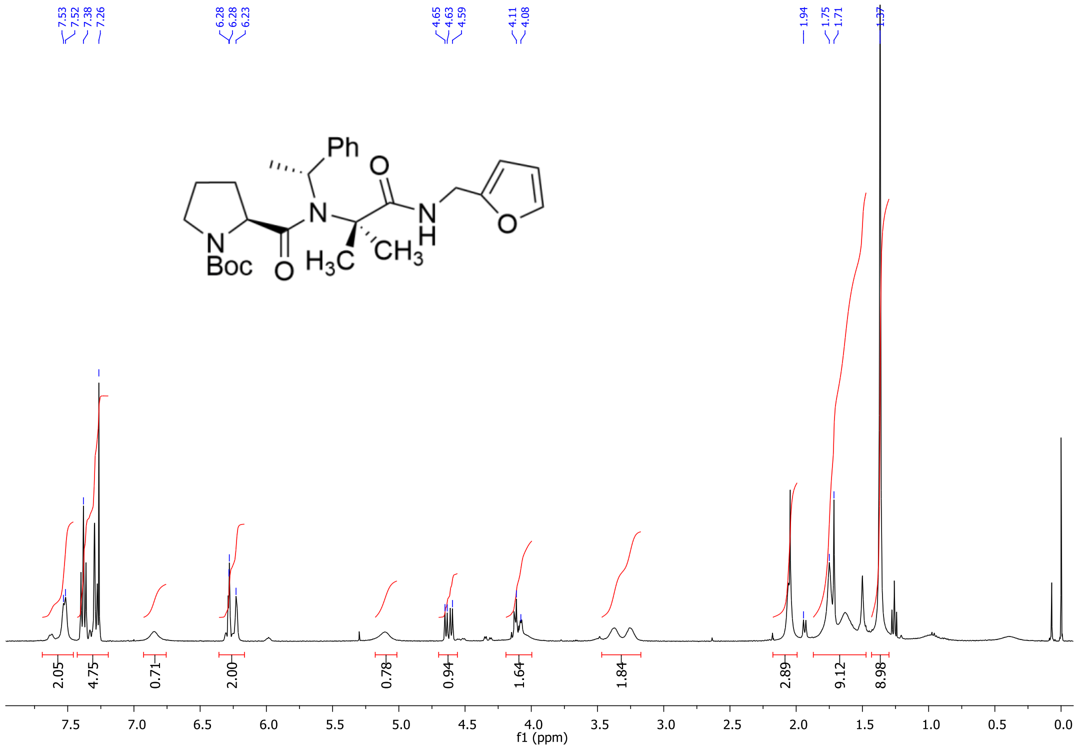

Figure S3: 400 MHz ^{1}H NMR spectrum in $CDCl_3$ of **2**.

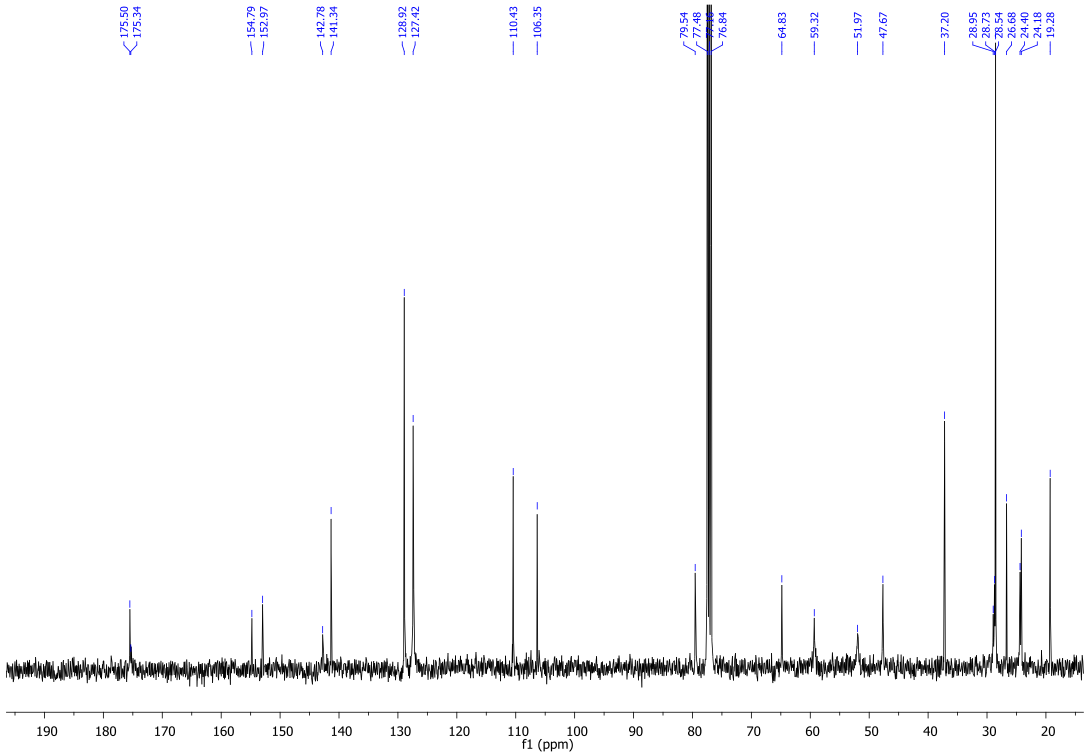

Figure S4: 100 MHz ^{13}C NMR spectrum in $CDCl_3$ of **2**.

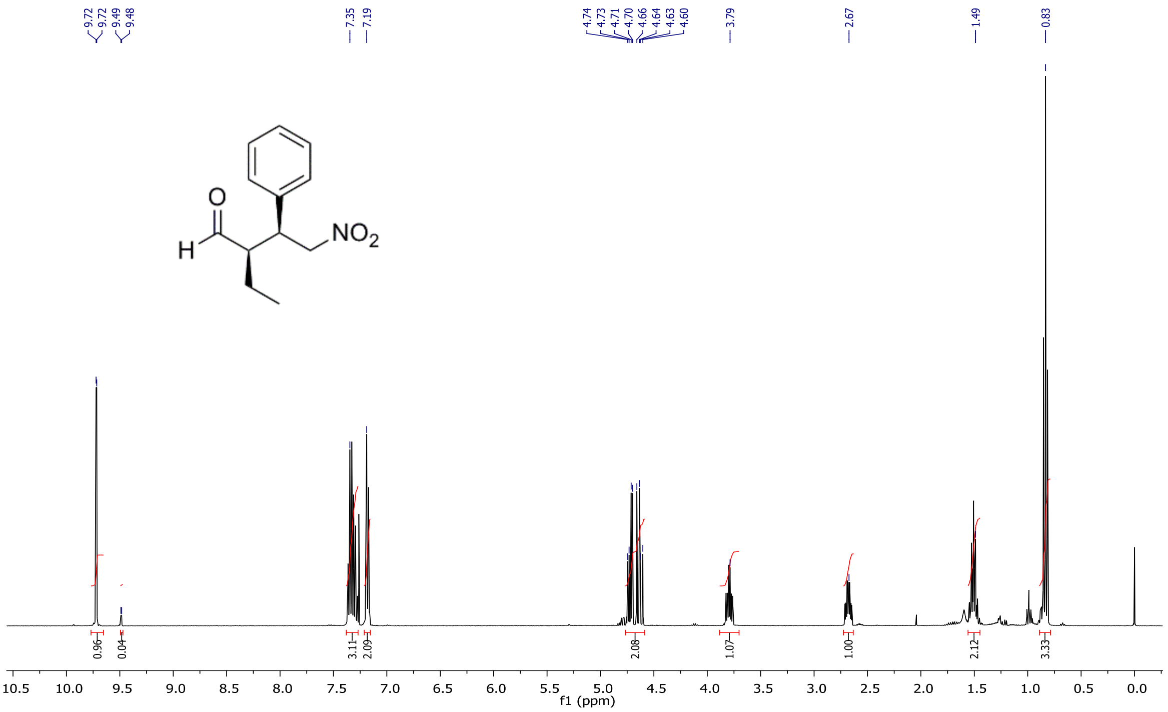

Figure S5: 400 MHz ^{1}H NMR spectrum in $CDCl_3$ of (2R,3S)-2-Ethyl-4-nitro-3-phenylbutanal (**5**).

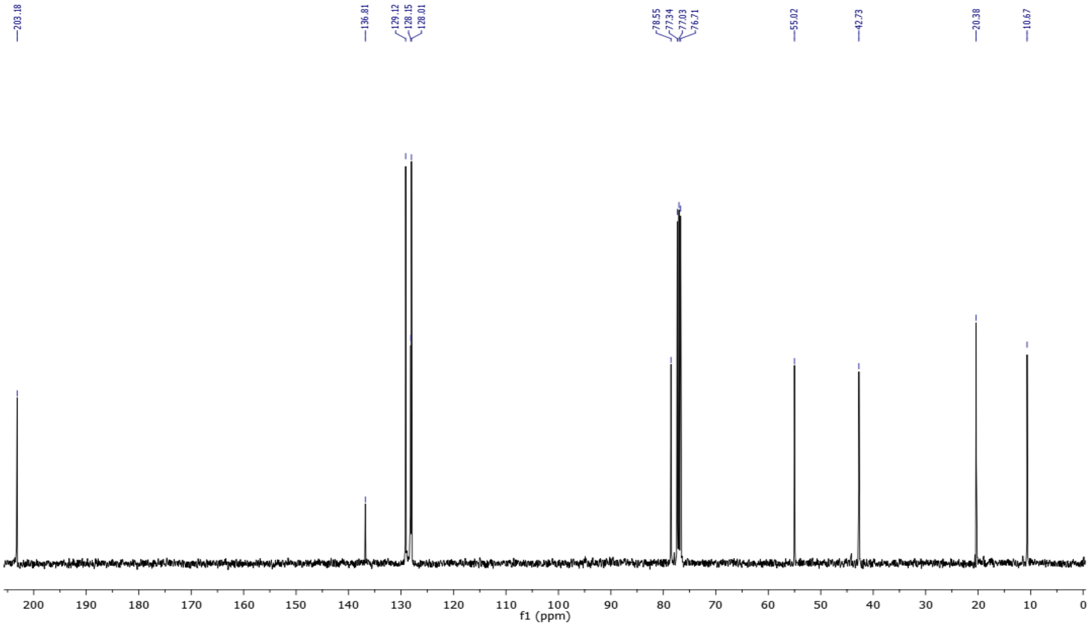

Figure S6: 100 MHz ^{13}C NMR spectrum in $CDCl_3$ of (2R,3S)-2-ethyl-4-nitro-3-phenylbutanal (**5**).

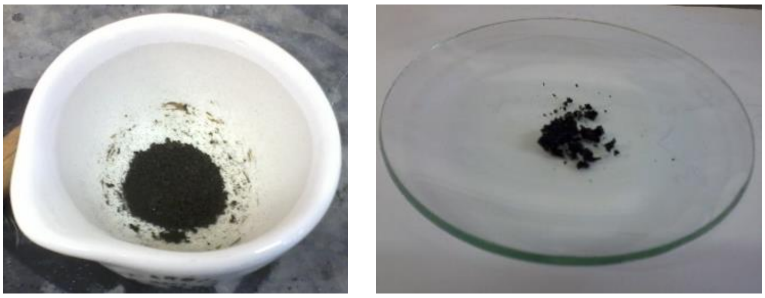

Figure S7: Photograph of PFA-supported catalysts material.

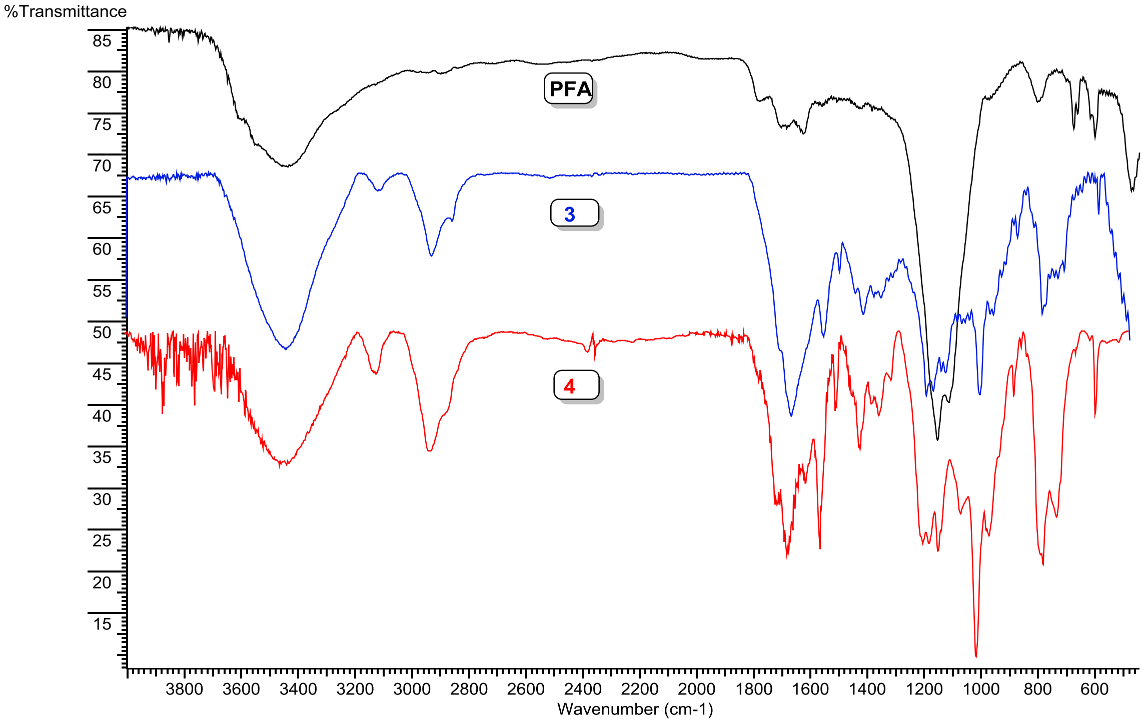

Figure S8: FT-IR spectra of polymers **PFA** (black), **3** (blue) and **4** (red) in the range of 4000–600 cm^{-1}.

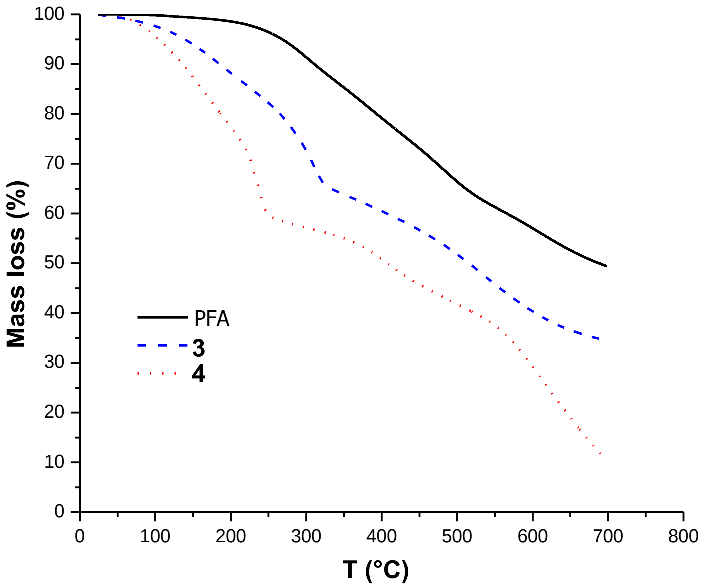

Figure S9: Variation of mass *versus* temperature measured by TGA for the **PFA**, **3** and **4** conducted under oxidative atmosphere at 10 °C min^{-1}.

Chiral HPLC results and spectra

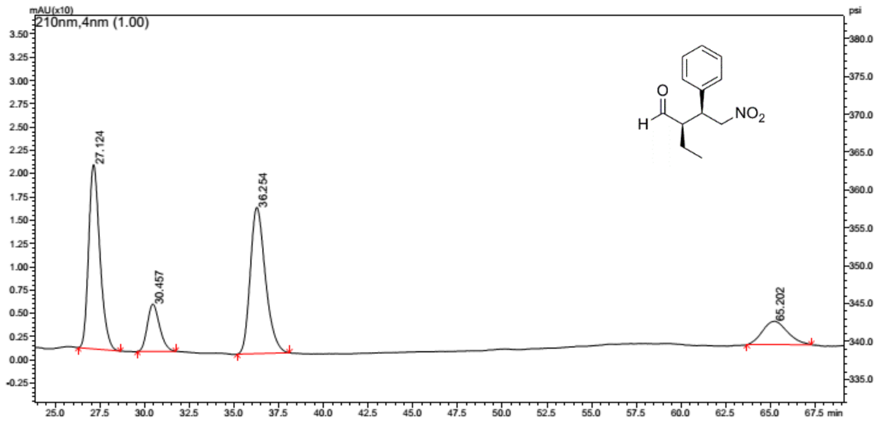

PeakTable

PDA Ch1 210nm 4nm

Peak#	Ret. Time	Area	Height	Area %	Height %
1	27.124	867445	19800	37.957	45.951
2	30.457	242098	5070	10.593	11.767
3	36.254	931040	15712	40.739	36.463
4	65.202	244769	2508	10.710	5.819
Total		2285353	43090	100.000	100.000

Figure S10: Chiral HPLC of racemic 2-ethyl-4-nitro-3-phenylbutanal. Chiralpak OD-H (*n*-hexane/iPrOH 91:9), 25 °C at 0.9 ml min^{-1}, UV detection at 210 nm.

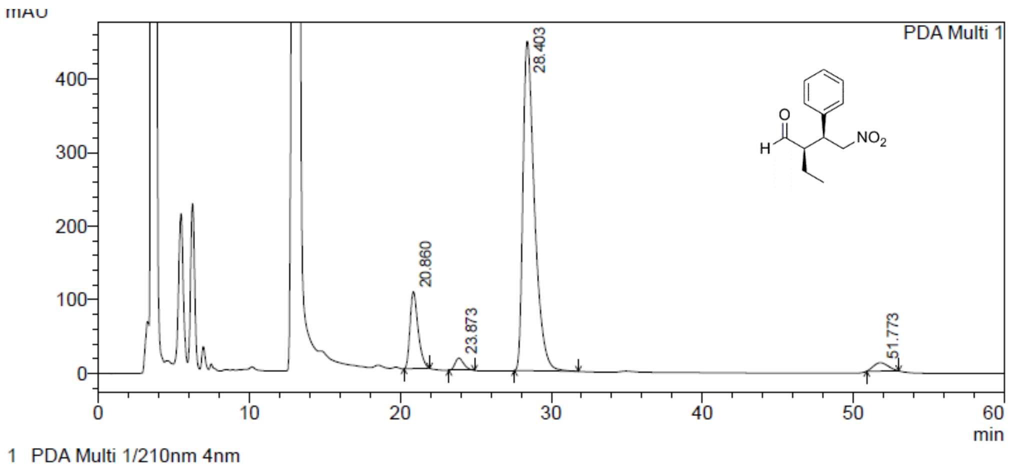

PeakTable

PDA Ch1 210nm 4nm

Peak#	Ret. Time	Area	Height	Area %	Height %
1	20.860	3875196	104361	13.278	17.997
2	23.873	674856	16601	2.312	2.863
3	28.403	23865884	447601	81.774	77.189
4	51.773	769271	11317	2.636	1.952
Total		29185207	579880	100.000	100.000

Figure S11: Chiral HPLC of the crude asymmetric 2-ethyl-4-nitro-3-phenylbutanal (**5**) obtained by bath reaction with PFA-supported catalyst **3**. Chiralpak OD-H (n-hexane/iPrOH 90:10), 25 °C) at 1.0 ml min^{-1}, UV detection at 210 nm of the crude reaction.

Table S1: Results of continuous flow Michael addition between *n*-butanal and β-nitrostyrene using the microreactor filled with PFA-supported catalyst **3**.

Entry [a]	Flow rate ϕ(µL min^{-1})	Running time (h)	Residence time τ(min)[b]	Conversion [c]	*dr.* (*syn:anti*) [e]	ee% [f]
1	2.5	0-10	-	-	-	-
2	2.5	10-12	140	25	95:5	74
3	2.5	12-14	140	27	95:5	75
4	2.5	14-16	140	30	95:5	74
5	2.5	16-18	140	31	95:5	73
6	2.5	18-20	140	38	95:5	74
7[d]	2.5	20-22	140	42	95:5	72
8	1	24-36	349	43	94:6	72
9	1	36-48	349	21	94:6	72
10	1	48-72	349	24	94:6	72

a) Reaction conditions: HPLC column (0.21 cm i.d. x 15 cm, containing 0.639 mmol of catalyst **3**); β-nitrostyrene (2.5 mmol, 1 equiv, 0.25 M), *n*-butanal (3 equiv, 0.75 M) in toluene. b) Residence time calculated as void volume/rate flow ($\boldsymbol{\tau = V_0/\phi}$). c) Conversion determined by ^{1}H NMR spectroscopy. d) Productivities are measured in mmol product h^{-1} mmol catalyst^{-1}. e) dr determined by ^{1}H NMR spectroscopy. f) Determined by chiral-stationary phase HPLC analysis.

Entry	HPLCs of Table 1.
2	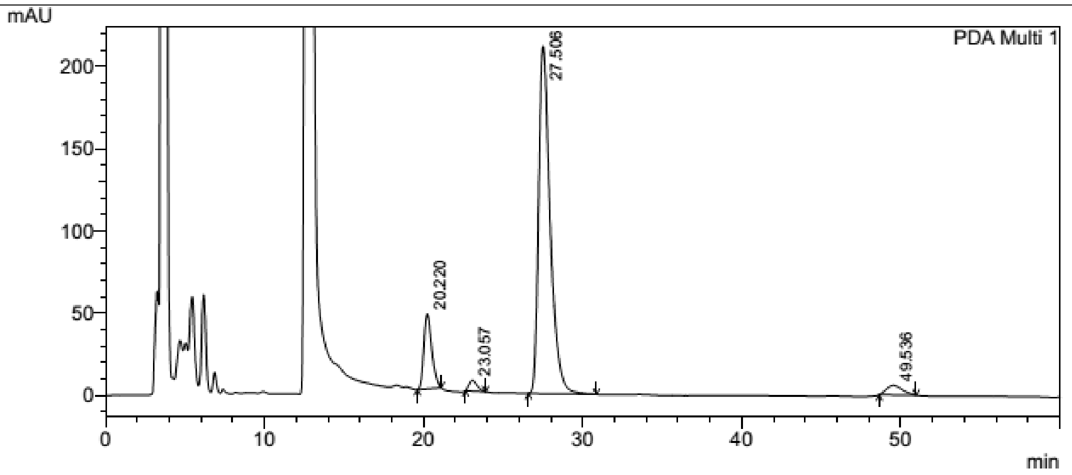

PeakTable

PDA Ch1 210nm 4nm

Peak#	Ret. Time	Area	Height	Area %	Height %
1	20.220	1587854	45517	12.317	16.913
2	23.057	229856	6527	1.783	2.425
3	27.506	10678706	211414	82.837	78.558
4	49.536	394748	5660	3.062	2.103
Total		12891163	269118	100.000	100.000

3

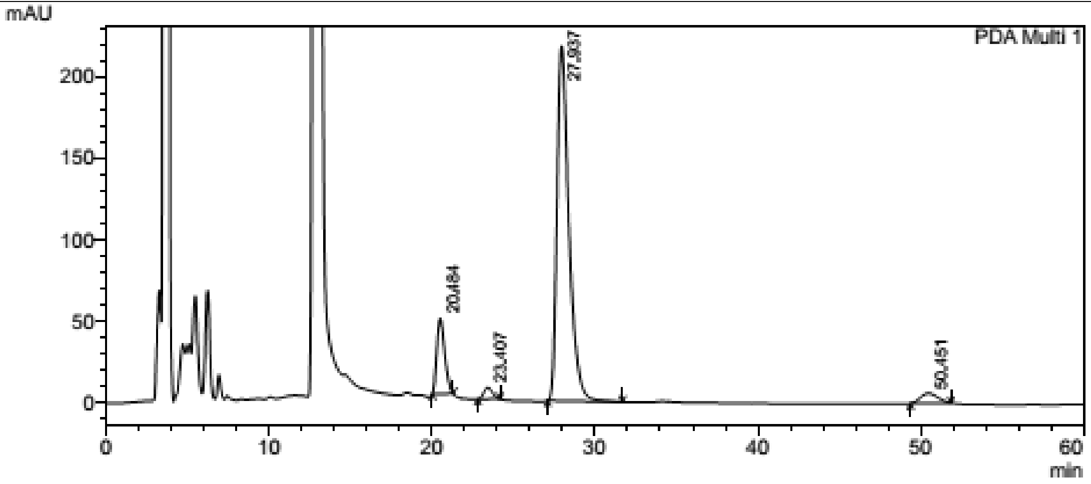

1 PDA Multi 1/210nm 4nm

PeakTable

PDA Ch1 210nm 4nm

Peak#	Ret. Time	Area	Height	Area %	Height %
1	20.484	1557976	46209	11.639	16.702
2	23.407	260434	7046	1.946	2.547
3	27.937	11116474	217305	83.047	78.543
4	50.451	450875	6108	3.368	2.208
Total		13385758	276669	100.000	100.000

4

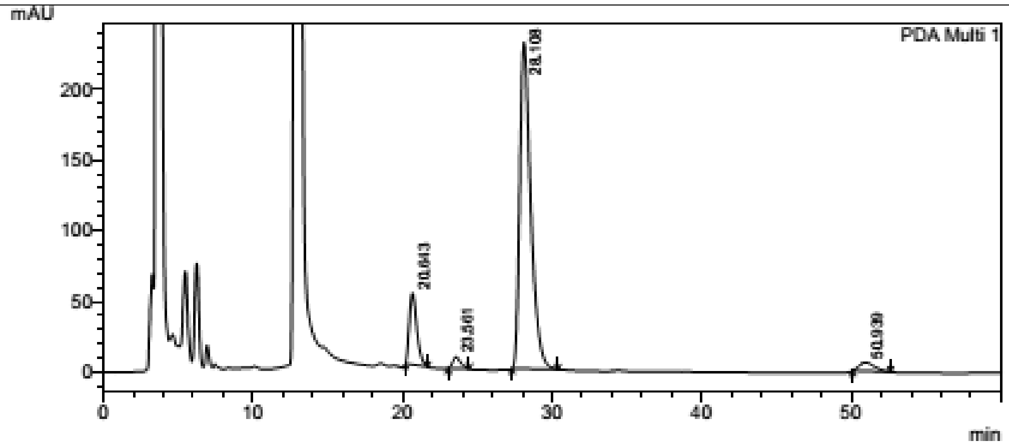

1 PDA Multi 1/210nm 4nm

PeakTable

PDA Ch1 210nm 4nm

Peak#	Ret. Time	Area	Height	Area %	Height %
1	20.643	1759063	50465	12.300	17.081
2	23.561	304022	7992	2.126	2.705
3	28.108	11808725	230951	82.570	78.168
4	50.939	429640	6046	3.004	2.046
Total		14301450	295454	100.000	100.000

5

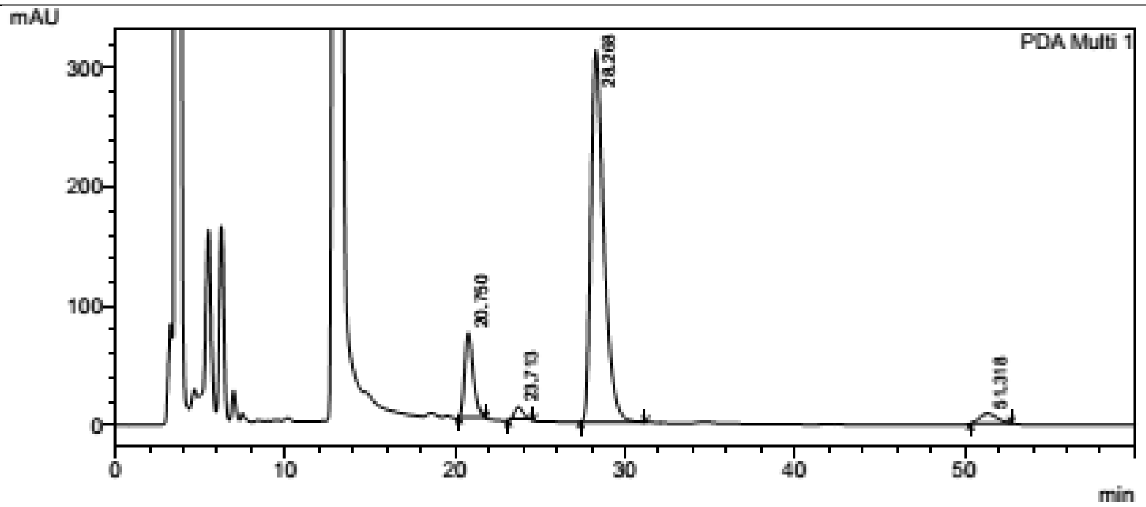

1 PDA Multi 1/210nm 4nm

PeakTable

PDA Ch1 210nm 4nm

Peak#	Ret. Time	Area	Height	Area %	Height %
1	20.750	2558251	71344	12.801	17.688
2	23.713	430531	11138	2.154	2.761
3	28.268	16396233	312604	82.044	77.501
4	51.318	599641	8266	3.001	2.049
Total		19984657	403353	100.000	100.000

6

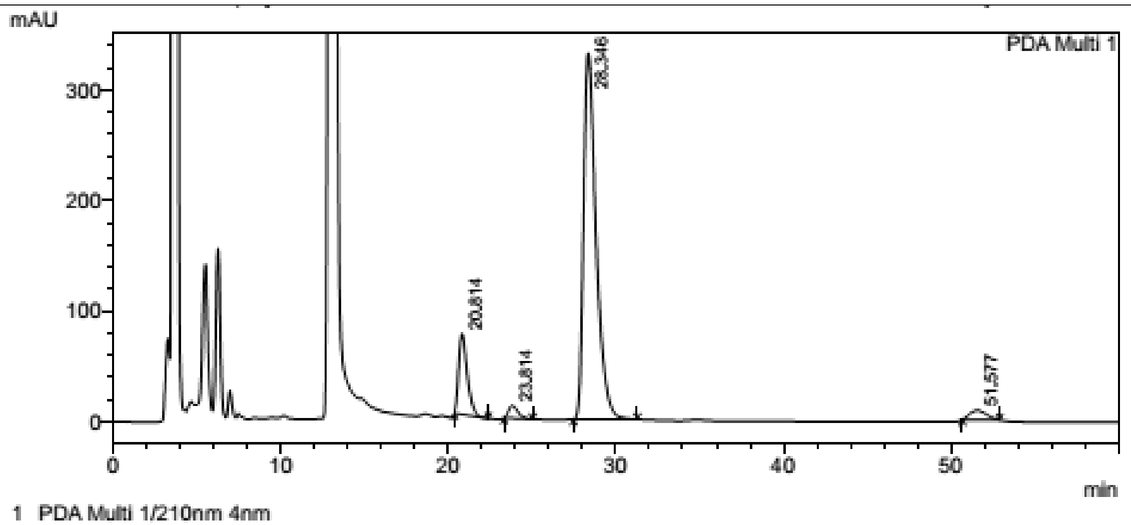

1 PDA Multi 1/210nm 4nm

PeakTable

PDA Ch1 210nm 4nm

Peak#	Ret. Time	Area	Height	Area %	Height %
1	20.814	2609783	72871	12.500	17.194
2	23.814	445781	11406	2.135	2.691
3	28.346	17230466	330988	82.526	78.095
4	51.577	592685	8561	2.839	2.020
Total		20878715	423826	100.000	100.000

7

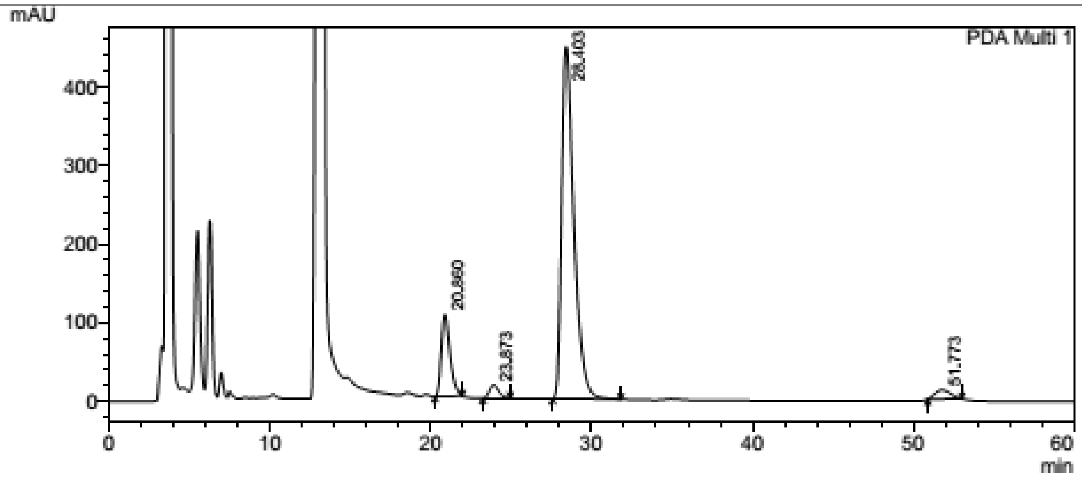

1 PDA Multi 1/210nm 4nm

PeakTable

PDA Ch1 210nm 4nm

Peak#	Ret. Time	Area	Height	Area %	Height %
1	20.860	3875196	104361	13.278	17.997
2	23.873	674856	16601	2.312	2.863
3	28.403	23865884	447601	81.774	77.189
4	51.773	769271	11317	2.636	1.952
Total		29185207	579880	100.000	100.000

8

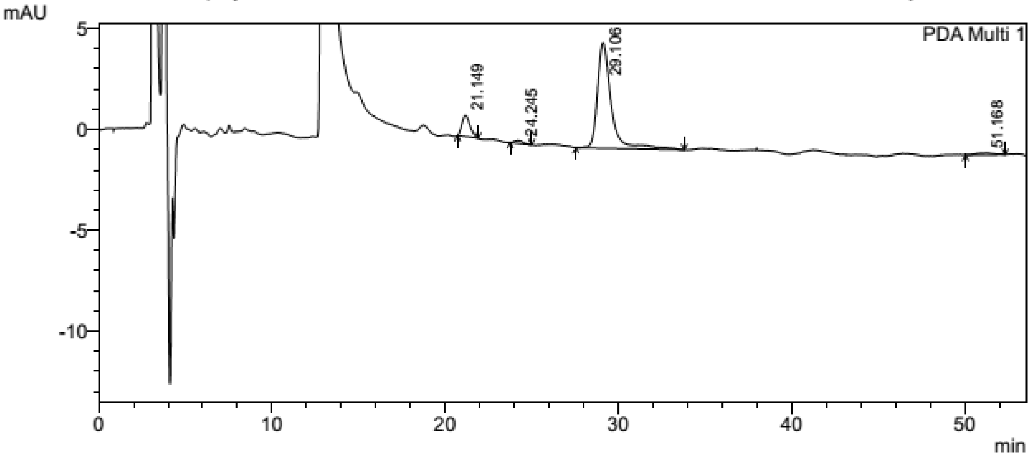

1 PDA Multi 1/210nm 4nm

PeakTable

PDA Ch1 210nm 4nm

Peak#	Ret. Time	Area	Height	Area %	Height %
1	21.149	34196	1054	10.236	16.098
2	24.245	4504	148	1.348	2.267
3	29.106	288448	5240	86.342	80.030
4	51.168	6929	105	2.074	1.606
Total		334078	6547	100.000	100.000

9

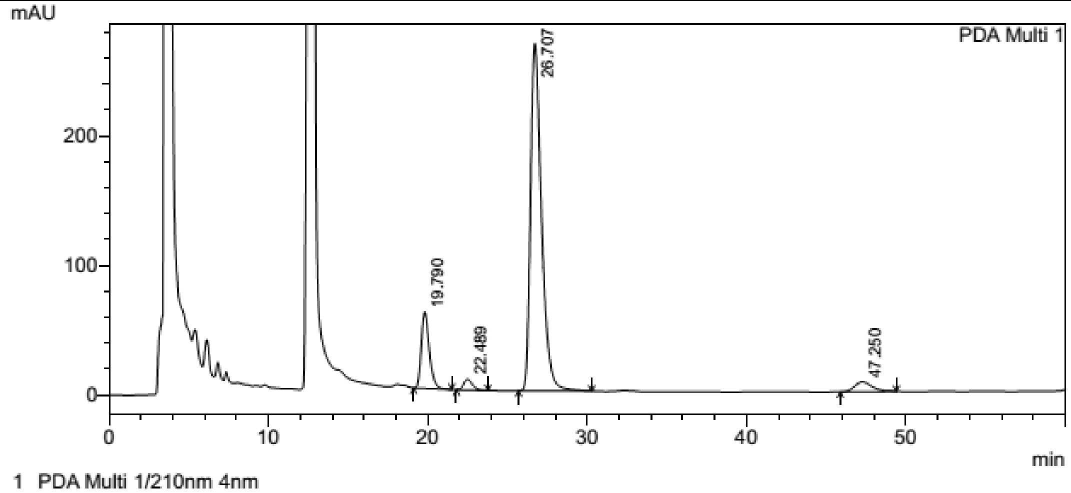

PeakTable

PDA Ch1 210nm 4nm

Peak#	Ret. Time	Area	Height	Area %	Height %
1	19.790	2045275	58935	13.030	17.215
2	22.489	308886	8179	1.968	2.389
3	26.707	12772745	267759	81.372	78.214
4	47.250	569814	7469	3.630	2.182
Total		15696720	342341	100.000	100.000

10

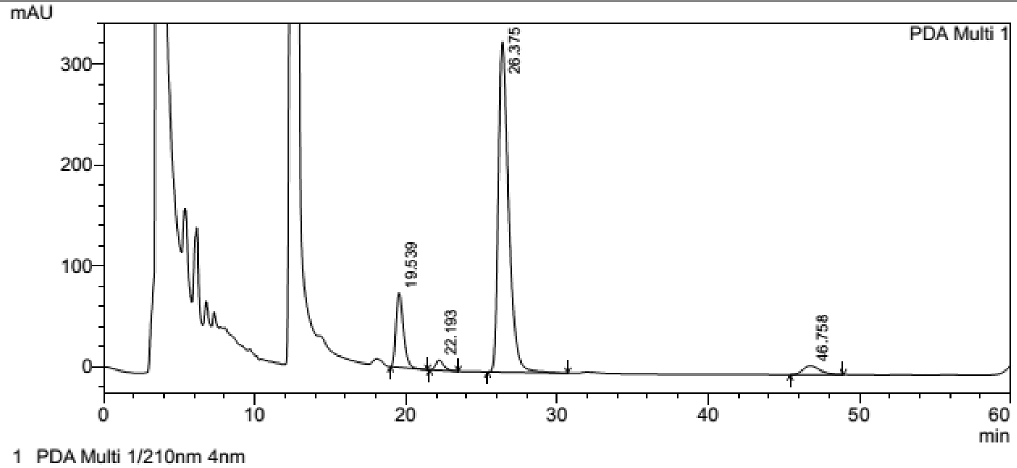

PeakTable

PDA Ch1 210nm 4nm

Peak#	Ret. Time	Area	Height	Area %	Height %
1	19.539	2514588	73878	13.158	17.609
2	22.193	373640	10131	1.955	2.415
3	26.375	15533770	326639	81.282	77.855
4	46.758	688913	8902	3.605	2.122
Total		19110912	419550	100.000	100.000

Figure S12: Different examples of chiral HPLC analysis of the crude asymmetric 2-ethyl-4-nitro-3-phenylbutanal (**5**) obtained by continuous flow Michael addition reaction with PFA-supported catalyst **3** **(**Table S1). Chiralpak OD-H (*n*-hexane/iPrOH 90:10), 25 °C, 1.0 ml min^{-1}, UV detection at 210 nm of the crude reaction.
